# Possible Biomarkers of Chronic Stress Induced Exhaustion - A Longitudinal Study

**DOI:** 10.1371/journal.pone.0153924

**Published:** 2016-05-04

**Authors:** Johanna Wallensten, Marie Åsberg, Åke Nygren, Robert Szulkin, Håkan Wallén, Fariborz Mobarrez, Anna Nager

**Affiliations:** 1 Division of Rehabilitation Medicine, Danderyd University Hospital, Stockholm, Sweden; 2 Department of Clinical Sciences, Karolinska Institutet, Danderyd Hospital, Stockholm, Sweden; 3 Department of Medical Epidemiology and Biostatistics, Karolinska Institutet, Stockholm, Sweden; 4 Academic Primary Healthcare Center, Stockholms läns landsting, Karolinska Institutet, Stockholm, Sweden; 5 Division of Cardiovascular Medicine, Danderyd Hospital, Stockholm, Sweden; 6 Department of Medicine, Rheumatology Unit, Karolinska University Hospital, Solna, 17176, Stockholm, Sweden; 7 Department of Neurobiology, Care Science and Society, Division of Family Medicine, Karolinska Institutet, Stockholm, Sweden; Istituto Superiore di Sanità, ITALY

## Abstract

**Background:**

Vascular endothelial growth factor (VEGF), epidermal growth factor (EGF) and monocyte chemotactic protein-1 (MCP-1) have previously been suggested to be potential biomarkers for chronic stress induced exhaustion. The knowledge about VEGF has increased during the last decades and supports the contention that VEGF plays an important role in stress and depression. There is scarce knowledge on the possible relationship of EGF and MCP-1 in chronic stress and depression. This study further examines the role of VEGF, EGF and MCP-1 in women with chronic stress induced exhaustion and healthy women during a follow-up period of two years.

**Methods and Findings:**

Blood samples were collected from 105 women with chronic stress induced exhaustion on at least 50% sick leave for at least three months, at inclusion (T0), after 12 months (T12) and after 24 months (T24). Blood samples were collected at inclusion (T0) in 116 physically and psychiatrically healthy women. The plasma levels of VEGF, EGF and MCP-1 were analyzed using Biochip Array Technology. Women with chronic stress induced exhaustion had significantly higher plasma levels of VEGF and EGF compared to healthy women at baseline, T12 and at T24. There was no significant difference in plasma levels of MCP-1. Plasma levels of VEGF and EGF decreased significantly in women with chronic stress induced exhaustion during the two years follow-up.

**Conclusions:**

The replicated findings of elevated levels of VEGF and EGF in women with chronic stress induced exhaustion and decreasing plasma levels of VEGF and EGF during the two years follow-up add important knowledge to the pathophysiology of chronic stress induced exhaustion.

## Introduction

In the late 1990’s, the number of people on long-term sick leave increased in Sweden, mainly due to stress-induced mental disorders. Many of these patients exhibited persistent fatigue and cognitive problems such as memory loss and lack of concentration. The Swedish National Board of Health and Welfare suggested that the term Exhaustion Disorder (ED) should be used for this fatigue condition apparently caused by prolonged stress without sufficient recovery (2003).

In 2004 the diagnosis ED was added to the Swedish version of the International Classification of Diseases, tenth version (ICD-10). Equivalents to ED in the international literature are chronic burnout [1], clinical burnout [2], stress-related exhaustion [3], job stress related depression [4,5] and possibly neurasthenia [6].

ED may occur after long-term stress of at least six months duration, according to the diagnostic criteria [7]. Characteristic symptoms are mental and physical fatigue, sleep disturbance, emotional problems such as irritability and depressed mood, cognitive problems and reduced stress tolerance (Table 1). The cognitive problems have been substantiated by psychological testing and involve impaired memory and reduced auditory and visual attention [1]. There is some evidence that chronic work-related stress may be associated with regional morphological changes in the brain [8,9]. Symptoms of depression and anxiety are often present at onset, but usually remit long before the fatigue and the cognitive problems [10,11] indicating that ED is a diagnostic entity different from depression.

**Table 1:** Criteria for Exhaustion Disorder according to the Swedish National Board of Health and Welfare^a^.

A. Physical and mental symptoms of exhaustion during at least two weeks. The symptoms have developed in response to one or more identifiable stressors present for at least six months.
B. The clinical picture is dominated by markedly reduced mental energy, as manifested by reduced initiative, lack of endurance, or increased time needed for recovery after mental effort.
C. At least four of the following symptoms have been present, nearly every day, during the same 2-week period:
1. Concentration difficulties or impaired memory
2. Markedly reduced capacity to tolerate demands or to work under time pressure
3. Emotional instability or irritability
4. Sleep disturbance
5. Marked fatigability or physical weakness
6. Physical symptoms such as aches and pains, palpitations, gastrointestinal problems, vertigo or increased sensitivity to sound
Physical symptoms such as aches and pains, palpitations, gastrointestinal problems, vertigo or increased sensitivity to sound
D. The symptoms cause clinically significant distress or impairment in occupational, social or other important respects.
E. The symptoms are not due to the direct physiological effects of a substance (e.g., a drug of abuse, a medication) or a physical illness/injury (e.g., hypothyroidism, diabetes, infectious disease).

^a^Criterion A-E must be fulfilled to diagnose ED

In order to find biomarkers for ED our research group previously assessed several biological mediators involved in stress response and inflammation in a group of women on prolonged sick leave due to mild mental illness [12]. The study demonstrated significantly elevated plasma concentrations of vascular endothelial growth factor (VEGF), epidermal growth factor (EGF) and monocyte chemotactic protein-1 (MCP-1) in women with prolonged sick leave due to mild mental illness, clinically diagnosed as exhaustion disorder. The levels of EGF were more than two times higher than in the healthy female controls and the levels of VEGF were three times higher, indicating that VEGF and EGF might be potential biomarkers for ED [12].

There is growing evidence that VEGF plays a role in stress and depression. Some studies have shown increased plasma levels of VEGF in patients with depression [13–17]. However, after adjusting for childhood trauma, the association between VEGF and depression were no longer significant according to one of these studies [14].

Another connection between depression and VEGF is the finding that VEGF stimulates the neurogenesis induced by antidepressant medication [18,19]. In rodents, chronic stress has been shown to reduce hippocampal volume in a process involving VEGF [20].

As far as we know, there is scarce knowledge about the possible relationship between EGF, stress and depression. A study on rhesus monkeys found that EGF seems to activate the hypothalamic-pituitary-adrenal axis (HPA axis) through stimulation of corticotropin-releasing hormone (CRH) release from the hypothalamus [21]. Interestingly, previous research has shown that the HPA axis is less sensitive to CRH in patients with ED compared to healthy controls [4,5].

There is also little knowledge about MCP-1 and its possible relationship to stress and depression. A study that examined vascular inflammation in rabbits found that unpredictable chronic mild stress resulted in increased aortic mRNA and protein expression of MCP-1 [22].

In order to further examine the role of plasma levels of VEGF, EGF and MCP-1 in patients with ED, we performed a follow-up study over two years on plasma levels of VEGF, EGF and MCP-1 in a new material of patients with ED and physically and psychiatrically healthy controls.

## Methods

### Patients with Exhaustion Disorder

Woman and men with psychiatric conditions (exhaustion disorder, burnout, neurasthenia, anxiety or depression) were selected consecutively from a database containing information about all public employees currently on long term sick leave (at least 50% sick leave for at least three months). This database is handled by one of the largest insurance companies in Sweden, AFA försäkring. Letters with an invitation to participate in the study were sent from the insurance company AFA försäkring. In the letter, individuals were informed that they were welcome to participate in the study if they considered their illness to be work-related. Individuals who did not reject further contact were contacted by the Karolinska Institute research group by telephone for detailed information about the study and for a telephone interview. The individuals lived in the Stockholm area and were 28 to 55 years old.

Individuals who were able to read and write the Swedish language were invited to a computerized Structured Clinical Interview for DSM-IV Axis 1 Disorders and Axis II Personality Disorders (SCID) [23,24] and a subsequent psychiatric and medical assessment performed by an experienced physician.

Those with abuse of alcohol and/or drugs, psychosis, anorexia or bulimia, bipolar disorder, severe personality disorder, serious neurological or endocrine disorder or other serious acute or chronic illnesses were excluded to ensure that the exhaustion was due to long term stress and no other disorder.

Individuals who fulfilled diagnostic criteria for ED (further called patients) were asked to participate. Diagnostic criteria for ED are shown in table 1. The diagnosis ED was only assessed at baseline.

### Healthy controls

The healthy controls were recruited five years after the patients, to serve as reference material to the patients. The Central Bureau of Statistics (SCB) randomly selected 1146 individuals, aged 28–55, from the population with permanent residence in the Stockholm area in the Swedish population register. Invitation letters for participation in the study were sent from SCB to the individuals in 2009. The individuals who agreed to participate were contacted for a first screening by phone. If they claimed to be healthy they were invited to a medical investigation including interview and clinical examination performed by an experienced physician. Individuals with current or previous ED, other types of mental illness, myocardial infarction, stroke or tumor disease were excluded.

### Descriptive characteristics

#### Educational level

Educational level was divided into three groups; examination from (1) elementary school (9 years of education), (2) upper secondary school (11–13 years of education) or (3) university (>13 years of education).

#### Current depression and anxiety disorders

Patients that fulfilled DSM IV criteria for depression at the baseline medical investigation were considered to have depression in addition to ED [25]. Patients that fulfilled DSM IV criteria for panic disorder with or without agoraphobia, social phobia, posttraumatic stress disorder or generalized anxiety disorder were considered to have an anxiety disorder in addition to ED [25].

#### Antidepressant medication

Patients who were on antidepressant medication at the baseline medical investigation were considered to have ongoing treatment with antidepressant medication.

#### Vascular disease

Patients were considered to have vascular disease if they had hypertension, angina pectoris or a history of myocardial infarction, congestive heart failure, or stroke.

### Analytical methods

Blood samples were obtained at inclusion (T0), after 12 months (T12) and after 24 months (T24) in patients and only at inclusion in controls. Blood samples were drawn through direct venipuncture from an antecubital vein after 15 minutes of rest. Patients in the present study were initially recruited for an intervention study five years before the controls. Blood sampling routines therefore differed slightly between patients and controls. The controls were instructed to refrain from food or drink for at least 12 hours, except for water, and all blood samples were taken in the morning. However, patients had no restrictions on intake of food or drink and 45% of the blood samples were drawn in the afternoon. Blood samples were collected into tubes containing sodium citrate and immediately centrifuged at 2000g for 20 minutes at room temperature. It was stored in -80°C until analyzed. Plasma levels of VEGF, EGF and MCP-1 from controls and from patients at baseline, after 12 months and after 24 months were analyzed with a commercially available biochip immunoassay system, Randox high sensitivity cytokine array (Randox Laboratories, Antrim, Northern Ireland, United Kingdom) [12]. Briefly, each biochip was coated with antibodies against VEGF, EGF and MCP-1. After 24 hours of incubation (including wash and addition of a secondary antibody) the samples were analyzed in the Randox investigator. All the values obtained in the present study were within the range of the standard/calibration curve. The samples were not run in duplicates. Plasma levels of VEGF, EGF and MCP-1 from controls and patients were analyzed in different years and therefor in different batches. The T0, T12 and T24 samples from each individual was analyzed in the same batch. The inter, intra-assay variation for the high-sensitive cytokine array was <10% according to manufacturer.

### Statistical analysis

STATA/IC 10 was used to perform statistical analyzes. Possible differences in age, educational level, plasma levels of VEGF, EGF and MCP-1 between ED-patients and controls were assessed using Mann-Whitney test, due to skewed data. Basic correlations between time from T0 until T24 and biomarkers (VEGF,EGF and MCP-1) were calculated using Spearman’s correlation. Spearman’s correlation was also used for basic correlations between age and biomarkers (VEGF,EGF and MCP-1).

A median regression analysis model was used to examine associations between outcome variables VEGF, EGF and MCP-1 and antidepressive medication, depression, anxiety, vascular disease or time of blood sampling (morning or afternoon) in patients.

In patients, a logistic regression model for repeated measurements was used to analyze if plasma levels of VEGF and EGF changed over time. Shapiro-Wilk test was used for test of normality. Since the outcomes of VEGF and EGF were strongly skewed the variables were dichotomized and a logistic Generalized Estimation Equation (GEE) model for repeated measurements with an unstructured correlation matrix was used to analyze change over time. The cut-off points were chosen based on the distribution of the variables rather than on clinical experience, since no clinical guidelines for high/low plasma levels exist. The highest plasma level for the controls (39 pg/ml for VEGF and 16 pg/ml for EGF) were chosen cut-off points.

For all statistical tests, p < 0.05 was considered statistically significant.

### Ethics

The study was approved by the Regional Ethical Review Board in Stockholm, Sweden, http://www.epn.se/en/start/ d.nr.2004/481-3, 2009/614-32 and 2014/585-31/1. All individuals gave their written informed consent.

## Results

**Fig 1:**
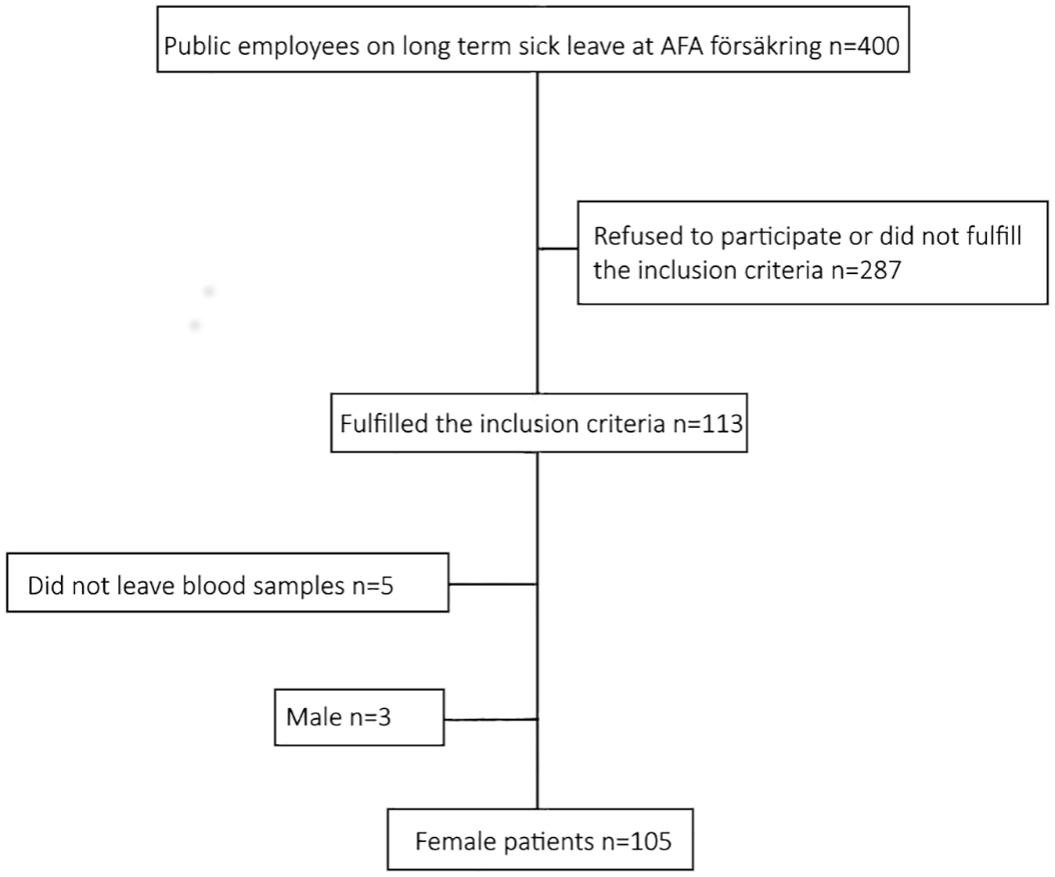
Flowchart of inclusion of patients.

Initially both men and women with ED were planned to be included in the study. However, only three men could be recruited. Therefore, we decided to include only women in the study. Inclusion criteria were met by 113 of the patients. After excluding all men (n = 3) and patients who did not leave blood sample (n = 5), 105 female patients with ED were finally included in the study (Fig 1).

**Fig 2:**
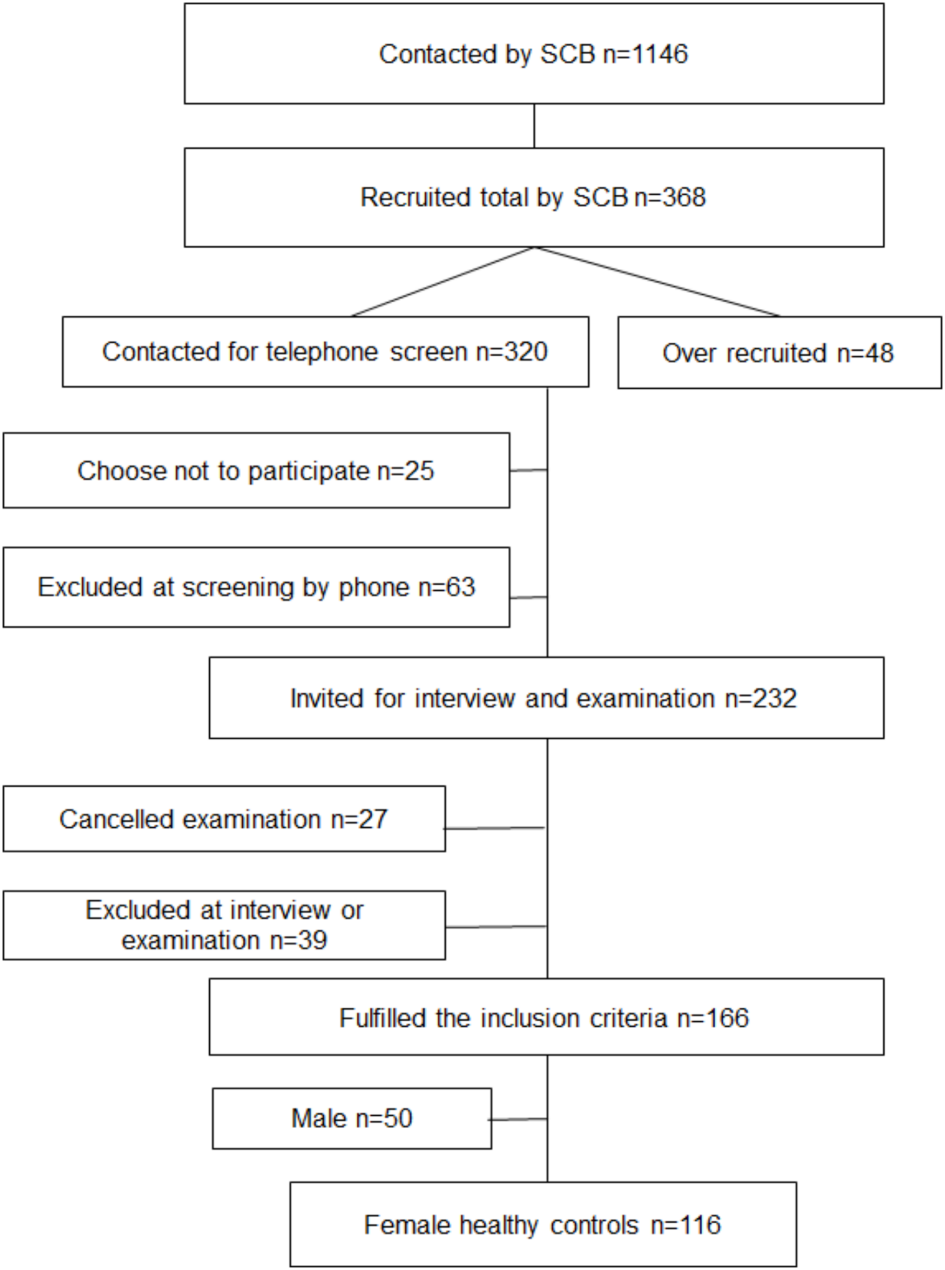
Flowchart of inclusion of controls.

A total of 368 healthy controls agreed to participate. Inclusion criteria were met by 166 of the controls. After excluding all men, 116 healthy female controls were finally included in the study (Fig 2).

Table 2 describes baseline characteristics of patients and controls. Age and educational level did not differ significantly between the groups.

**Table 2:** Descriptive characteristics of patients and controls.

Descriptive characteristic	Patients (n = 105)	Controls (n = 116)	P-value
**Age, mean (standard deviation)**	44.8^a^ (6.9)	45.3 (7.2)	0.446
**Educational level, median (25%-75%)**	3^b^ (2–3)	3 (2–3)	0.203
**Percent with antidepressant medication**	45.2%^c^	0%	-
**Percent with current depression**	59.6% ^c^	0%	-
**Percent with anxiety disorders**	17.1%	0%	-
**Percent with vascular disease**^d^	14.3%	0%	-
**Percent on full time sick-leave**	69.3% ^d^	0%	-
**Percent on part time sick-leave**	30.7% ^d^	0%	-

^a^3 missing values

^b^13 missing values

^c^1 missing value

^d^4 missing values

**Fig 3:**
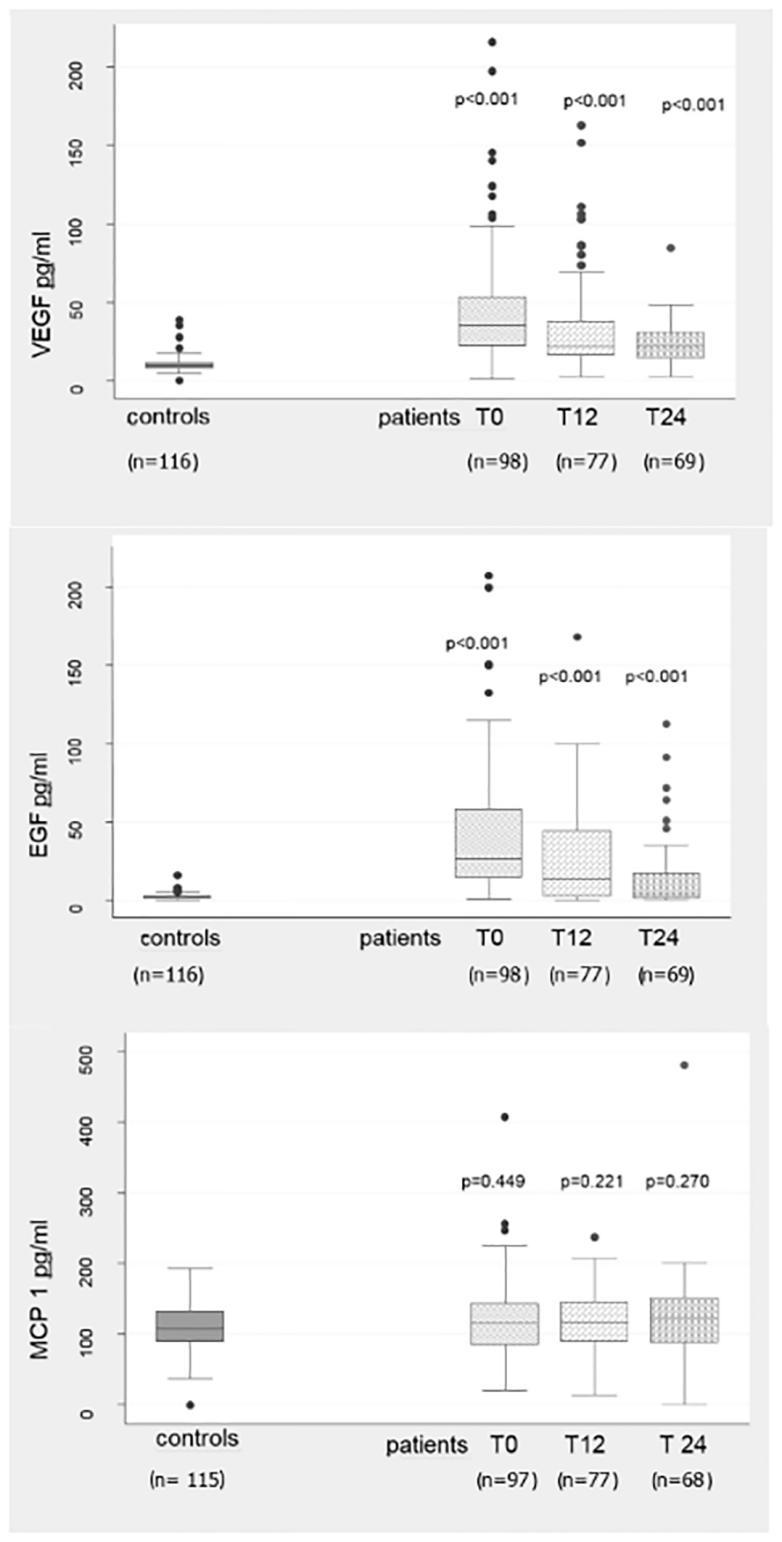
Box plots for plasma levels of VEGF, EGF and MCP-1 in controls and patients. Box plot for plasma levels of VEGF, EGF and MCP-1 in controls and patients at baseline (T0), after 12 months (T12) and after 24 months (T24). P-values refer to plasma levels of VEGF, EGF and MCP-1 in controls compared to time of follow up (T0, T12 or T24) in patients.

Table 3 shows plasma levels of VEGF, EGF and MCP-1 at baseline. Plasma levels of VEGF and EGF were significantly higher in patients compared to controls. There was no significant difference in MCP-1 between patients and controls. At baseline, plasma levels of VEGF and EGF in patients were widely spread with high standard deviations compared to controls. This is also shown in fig 3.

**Table 3:** Plasma levels of VEGF, EGF and MCP-1 in patients and controls at baseline.

	Patients (n = 105)		Controls (n = 116)		
	Mean (SD)	Median (Q1,Q3)	Mean (SD)	Median (Q1,Q3)	P-value
**VEGF (pg/ml)**	50.83 ^a^ (56.43)	35.24 ^a^ (22.45, 54.22)	10.35 (5.03)	9.48 (7.57, 11.53)	<0.001
**EGF (pg/ml)**	41.43 ^a^ (41.05)	26.77 ^a^ (14.36, 58.65)	2.33 (2.04)	2.16 (1.35, 3.04)	<0.001
**MCP-1 (pg/ml**)	122.37 ^b^ (55.67)	116.04 ^b^ (85.71, 143.70)	112.49 ^c^ (35.94)	107.20 ^c^ (89.90, 132.90)	0.45

^a^ 7 missing value

^b^ 8 missing values

^c^ 1 missing value

Table 4 shows that antidepressant medication, depression, anxiety, vascular disease or time of blood sampling (morning or afternoon) did not affect plasma levels of VEGF, EGF or MCP-1 in patients.

**Table 4:** Median regression of possible confounders for plasma levels of VEGF, EGF and MCP-1 at baseline (T0) in women with exhaustion disorder.

**VEGFT0**	**Coef.**	**Std. Err.**	**t**	**P>|t|**	**95% Conf. Interval**
Antidepressive medication	8.06	5.96	1.35	0.18	-3.78, 19.90
Depression	-0.33	6.91	-0.05	0.96	-14.07, 13.41
Anxiety disorders	-9.54	12.05	-0.79	0.43	-33.51, 14.43
Vascular disease	2.20	9.15	0.24	0.81	-15.99, 20.39
Time of bloodsampling	-1.22	5.98	-0.20	0.84	-13.12, 10.68
**EGFT0**	**Coef.**	**Std. Err.**	**t**	**P>|t|**	**[95% Conf. Interval]**
Antidepressive medication	9.17	8.44	1.09	0.28	-7.62, 25.96
Depression	-1.06	8.19	-0.13	0.90	-17.34, 15.22
Anxiety disorders	-3.30	19.31	-0.17	0.87	-41.70, 35.10
Vascular disease	-17.25	21.04	-0.82	0.42	-59.09, 24.59
Time of bloodsampling	-1.06	8.00	-0.13	0.90	-16.97, 14.85
**MCP-1T0**	**Coef.**	**Std.Err.**	**t**	**P>|t|**	**[95% Conf. Interval]**
Antidepressive medication	7.77	13.09	0.59	0.55	-18.25, 33.79
Depression	1.85	13.83	0.13	0.89	-25.64, 29,34
Anxiety disorders	-24.22	19.71	-1.23	0.22	-63.41, 14.97
Vascular disease	22.76	30.10	0.76	0.45	-37.08, 82.60
Time of bloodsampling	1.42	14.05	0.10	0.92	-26.52, 29.36

The plasma levels of VEGF and EGF decreased significantly with time among patients during the follow-up (p = <0.001 for both VEGF and EGF) (Table 5). However, the plasma levels of VEGF and EGF were still significantly higher in patients, both at 12 months and 24 months after inclusion, compared to controls at baseline (Fig 3).

**Table 5:** Summary Generalized Estimation Equation (GEE) model.

				GEE model^a^	
EGF	Time	OBS	Above cut-off, N(%)	Odds ratio (95% CI)	P-value
	0	77	52 (68)	-	-
	12	73	36 (49)	-	-
	24	65	19 (29)	0.94 (0.91–0.96)^b^	<0.001
**VEGF**	0	77	31 (40)	-	-
	12	73	18 (25)	-	-
	24	65	6 (9)	0.93 (0.90–0.96)^c^	<0.001

^a^Time was assumed to be linear in the GEE model. This assumption was assessed with the NLCHECK command in Stata.

^b^Interprets as: for every year the odds of being above the EGF-cutoff decrease with 6%.

^c^Interprets as: for every year the odds of being above the VEGF-cutoff decrease with 7%

Spearman’s correlation showed no significant correlation between age and VEGF (Spearman’s rho = -0.072, p = 0.30) or between age and EGF (Spearman’s rho = -0.12, p = 0.093). There was a significant correlation between age and MCP-1 (Spearman’s rho = 0.33, p = <0.001) (data not shown).

Spearman’s correlation showed significantly negative correlation between time (T0 until T24) and VEGF (Spearman’s rho = -0.32, p = <0.001) and EGF (Spearman’s rho = -0.41, p = <0.001 but not for MCP-1 (Spearman’s rho = 0.023, p = 0.77).

## Discussion

This follow-up study replicates previous findings showing that plasma levels of VEGF and EGF are increased in women with ED compared to healthy controls (Åsberg et al., 2009). In contrast, plasma levels of MCP-1 did not differ between women with ED and healthy controls in our material. Plasma levels of VEGF and EGF decreased significantly during the two year follow-up.

### Plasma levels of VEGF and EGF in ED in comparison with other studies

In two previous studies by Åsberg et al (2009) and Jonsdottir et al (2009), the levels of VEGF, EGF and MCP-1 in patients with ED were examined. These two studies have reached contradictory results, although materials and methods were similar. Åsberg et al. found that VEGF, EGF and MCP-1 were significantly increased in women with ED compared to healthy women. The study also indicated a “dose-response” effect, since an additional group of women with occupational stress had levels of VEGF, EGF and MCP-1 that were in between those of women with ED and healthy women. However, Jonsdottir et al, who used a similar group of women and the same analytic method as Åsberg et al, did not find increased plasma levels of VEGF, EGF and MCP-1 in women with ED compared to healthy controls [26].

A reason for different results in the studies could be a dissimilarity between the populations. There might be a difference in severity of ED and in residual confounding among the study populations. A possible confounder such as time from onset of ED to inclusion in study was not measured in any of the studies.

### Plasma levels of VEGF and EGF as biomarkers for ED

Åsberg et al proposed cut-off levels for VEGF, EGF and MCP-1 in plasma for screening and/or diagnostic purpose in ED [12]. Jonsdottir et al opposed to this since they found different levels of VEGF, EGF and MCP-1 using two different analytic techniques on their material [26]. In humans, VEGF family comprises of several growth factors such as VEGF (VEGF-A), VEGF-B, VEGF-C, VEGF-D, and placenta growth factor (PlGF) [27,28]. Unfortunately, the Randox analytic technique used in the present study does not disclose whether only VEGF-A, or also other types of VEGF are detected. Measuring different types of VEGF by different analytic methods could be a reason why Jonsdottir obtained other results.

In our study we found no correlation between age and levels of VEGF and EGF. This is in line with a previous study of VEGF [29]. However, we did find a correlation between MCP-1 and age, which is not in line with previous studies [30,31].

Before using a biomarker in clinical practice several replications of the results are needed. For VEGF and EGF analyses to be clinically useful, more knowledge on at least four areas is required. Firstly, an analytic method that distinguishes between different types of VEGF should be used. Secondly, since plasma levels of VEGF and EGF seem to decrease with time, plasma levels of VEGF and EGF may be correlated to time of onset of ED or to severity of symptoms. Thirdly, the effect of body mass index, intake of food or drink before blood sampling and possibly childhood trauma on plasma levels of VEGF and EGF in ED must be further examined. Finally, the potential for adding measures of vascular function should be examined.

### Plasma levels of VEGF and EGF as possible mediators of cardiovascular disease

An area for future studies is whether plasma levels of VEGF and EGF are possible mediators of cardiovascular disease in individuals with stress related disorders. Previous research has demonstrated an association between concentrations of VEGF and EGF and hypertension, compared with healthy individuals [32]. A Swedish register study has shown an increased risk of cardiovascular disease in individuals on long term sick-leave due to psychiatric disorder [33]. According to data from the Swedish social insurance office, about half of individuals on long term sick leave due to psychiatric problems suffer from stress related disorders [34]. VEGF and EGF may play a role in increased risk of cardiovascular disease in these individuals.

### Strengths and limitations

Replicated findings of a significant elevation of biomarkers for mental illness, as in this study, are rare. The findings are strengthened by the use of a carefully selected and thoroughly examined healthy control group with no current or previous history of mental illness, cardiovascular disorder or cancer.

The main limitations of the study are that controls and patients were collected in different years, that there were some differences in the conditions at blood sampling between the groups and that the plasma levels of VEGF, EGF and MCP-I were analyzed in different batches. These limitations may have affected the results. However, the regression analysis including time of blood sampling (morning or afternoon) could not explain differences in plasma levels of VEGF and EGF. In addition, a previous study on diurnal variation of VEGF, suggests that samples should be drawn after 7 am [29], which was done in both patients and controls in our study. Storage time has not been shown to correlate to the levels of VEGF in plasma stored in -80°C until analyzed [35]. We have not found data from previous studies about sampling factors that could affect the levels of EGF or MCP-1. In our study plasma levels of VEGF and EGF appeared to decrease during follow up, indicating that there is an association that could not only be explained by different sampling methods in controls and patients. Unfortunately, we have no follow up in controls, therefore we do not know if the levels of VEGF, EGF and MCP-1 changes over time in controls.

Other limitations are that the material did not allow for adjustment of nicotine status, hormonal status or body mass index.

## Conclusion

The replication of high levels of VEGF and EGF in ED compared to healthy controls, and the decrease in plasma levels of VEGF and EGF within two years, adds important knowledge to the pathophysiology of ED. The study calls for further research in order to develop accurate analytical methods for possible use of VEGF and EGF as biomarkers for ED.
